# Renal Function Underpins the Cyclooxygenase-2: Asymmetric Dimethylarginine Axis in Mouse and Man

**DOI:** 10.1016/j.ekir.2023.03.014

**Published:** 2023-03-23

**Authors:** Plinio Ferreira, Ricky Vaja, Maria Lopes-Pires, Marilena Crescente, He Yu, Rolf Nüsing, Bin Liu, Yingbi Zhou, Magdi Yaqoob, Anran Zhang, Matthew Rickman, Hilary Longhurst, William E. White, Rebecca B. Knowles, Melissa V. Chan, Timothy D. Warner, Elizabeth Want, Nicholas S. Kirkby, Jane A. Mitchell

**Affiliations:** 1 National Heart and Lung Institute, Imperial College London, United Kingdom; 2 Blizard Institute, Barts, and The London School of Medicine and Dentistry, London, United Kingdom; 3 Deparment of Pharmacology, Medical Sciences Division, University of Oxford, Oxford, United Kingdom; 4 Clinical Pharmacology and Pharmacotherapy Department, Goethe University, Frankfurt, Germany; 5 Cardiovascular Research Center, Shantou University Medical College, Shantou, China; 6 Department of Medicine, University of Auckland, and Department of Immunology, Auckland City Hospital, Auckland, New Zealand; 7 Department of Metabolism, Digestion and Reproduction, Imperial College London, London, United Kingdom; 8 Department of Life Sciences, Manchester Metropolitan University, Manchester, United Kingdom

**Keywords:** ADMA, kidney, nitric oxide, NSAID, prostacyclin

## Abstract

**Introduction:**

Through the production of prostacyclin, cyclooxygenase (COX)-2 protects the cardiorenal system. Asymmetric dimethylarginine (ADMA), is a biomarker of cardiovascular and renal disease. Here we determined the relationship between COX-2/prostacyclin, ADMA, and renal function in mouse and human models.

**Methods:**

We used plasma from COX-2 or prostacyclin synthase knockout mice and from a unique individual lacking COX-derived prostaglandins (PGs) because of a loss of function mutation in cytosolic phospholipase A_2_ (cPLA_2_), before and after receiving a cPLA_2_-replete transplanted donor kidney. ADMA, arginine, and citrulline were measured using ultra-high performance liquid-chromatography tandem mass spectrometry. ADMA and arginine were also measured by enzyme-linked immunosorbent assay (ELISA). Renal function was assessed by measuring cystatin C by ELISA. ADMA and prostacyclin release from organotypic kidney slices were also measured by ELISA.

**Results:**

Loss of COX-2 or prostacyclin synthase in mice increased plasma levels of ADMA, citrulline, arginine, and cystatin C. ADMA, citrulline, and arginine positively correlated with cystatin C. Plasma ADMA, citrulline, and cystatin C, but not arginine, were elevated in samples from the patient lacking COX/prostacyclin capacity compared to levels in healthy volunteers. Renal function, ADMA, and citrulline were returned toward normal range when the patient received a genetically normal kidney, capable of COX/prostacyclin activity; and cystatin C positively correlated with ADMA and citrulline. Levels of ADMA and prostacyclin in conditioned media of kidney slices were not altered in tissue from COX-2 knockout mice compared to wildtype controls.

**Conclusion:**

In human and mouse models, where renal function is compromised because of loss of COX-2/PGI_2_ signaling, ADMA levels are increased.

## Graphical abstract

**Figure:**
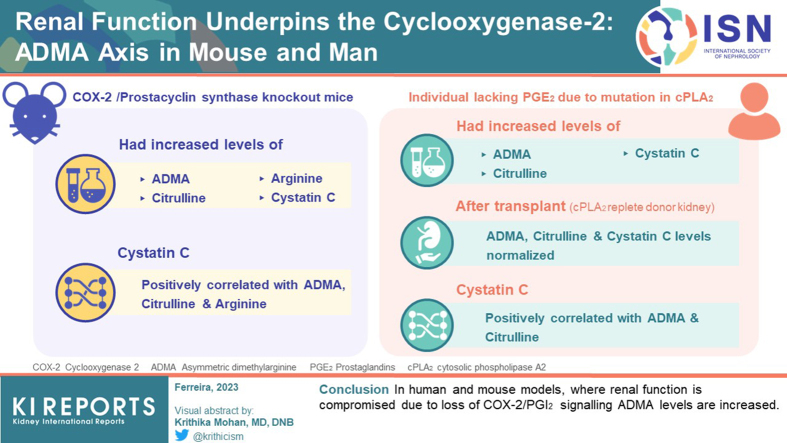

Nonsteroidal anti-inflammatory drugs (NSAIDs) work by blocking prostanoids produced by the inducible enzyme COX-2. COX-2 is also expressed constitutively in various anatomic locations^1^ where, among other functions, it protects the cardiovascular system.^2^ Although the location(s) of cardioprotective COX-2 and the associated mechanisms remain unclear,^3^ the kidney^2^^,^^4^^,^^5^ and areas of the vasculature^6^ have been suggested as important. In the kidney, COX-2 is localized to the medulla region^1^^,^^4^ within interstitial cells (fibroblast-like),^7^^,^^8^ where its activity regulates salt and water homeostasis, salt sensitive hypertension, papillary integrity, and apoptosis.^7^ In addition, in a systematic analysis of regional blood flow, we found that of the regions where COX-2 is expressed constitutively, blood flow was only reduced by acute COX-2 inhibition in the kidney.^9^ In line with this, of the COX-products, prostacyclin serves as a local vasodilator and protects against ischemia and fibrosis.^10^ PGE_2_ can also serve as a vasodilator in the kidney although its pharmacology is more complex,^11^ and PGE_2_ can contribute to renal dysfunction in some settings.^11^

As a consequence of the protective role of COX-2 in the kidney, NSAIDs increase the risk of renal compromise and hypertension particularly in those with underlying renal stress.^12^ NSAIDs also increase the risk of heart attacks and strokes,^13^ which may (in part) be directly or indirectly explained by the proatherogenic and prothrombotic environment associated with renal dysfunction.^14^ These features can be recapitulated in mice in as much as pharmacologic blockade and/or genetic deletion of COX-2 reduces renal function,^15^, ^16^, ^17^ elevates blood pressure,^18^ increases atherosclerosis,^19^ and exacerbates thrombosis^18^^,^^20^; although some of these effects are dependent on dose or type of NSAID, duration of treatment, and/or genetic background.^21^

Our work has suggested a link between inhibition of COX-2-derived prostacyclin in the kidney and increases in the methylarginine, ADMA.^17^^,^^22^ ADMA is formed when arginine residues in proteins are methylated by protein arginine methyltransferase enzymes and proteins are subsequently broken down.^22^^,^^23^ Although ADMA may be formed in all cells, the kidney is a prime site for generation, metabolism, and excretion of methylarginines. ADMA is a natural inhibitor of the cardioprotective enzyme endothelial nitric oxide synthase (eNOS)^24^ and therefore, events in the kidney resulting in increased ADMA levels may reduce renal and systemic endothelial function at the level of eNOS.^25^^,^^26^ In line with this, ADMA is a biomarker of renal dysfunction^27^ and of cardiovascular risk and all-cause mortality.^28^^,^^29^ Furthermore, Ricciotti *et al.*^30^ showed that in rodent models where COX-2 was inhibited or knocked out postnatally, renal function, blood pressure, and plasma ADMA levels remained normal but that ADMA increased in line with blood pressure and creatinine in mice treated with angiotensin II; and that reduced renal function correlated with ADMA in normotensive and hypertensive mice treated with or without the NSAID naproxen.

It is therefore likely that the link between COX-2 and ADMA is in whole^30^ or in part^17^^,^^22^ driven by reciprocal effects on renal function and that*,* increases in ADMA associated with loss of COX-2 would be directly related to renal dysfunction.

To further our understanding in this area, in the current study we have used samples from COX-2^17^^,^^31^ and prostacyclin synthase (PGIS) knockout mice^17^ to compare plasma levels of ADMA with the renal function marker cystatin C. To understand the relationship between renal function and ADMA in a human model and the kidney specifically, we have used samples from a patient with inherited human group IV A (cytosolic phospholipase A_2_) cPLA_2α_ deficiency.^32^ Because cPLA_2α_ is responsible for the liberation of arachidonic acid (substrate) for COX-2, this patient displayed an almost complete lack of prostanoid synthetic capacity.^32^^,^^33^ The patient subsequently underwent a kidney transplant receiving a normal (cPLA_2_ sufficient) organ, which restored the patient’s ability to produce prostanoids in the kidney but not elsewhere in the body.^34^ Using samples from this patient before and after the kidney transplant, we have been able to determine directly the contribution of kidney COX activity to renal function and circulating levels of ADMA. Because ADMA is derived from arginine and arginine cycles with citrulline, we have also reported levels of arginine and citrulline in plasma samples analyzed in this study. Finally, to delineate the effects of COX-2 deletion on synthetic capacity *in vitro* from renal function *in vivo*, we also measured ADMA from mouse organotypic kidney slices in culture.

## Methods

### Human Samples

This study utilizes samples from a patient with a homozygous 4 bp deletion (g.155574_77delGTAA) in the *PLA2G4A* gene resulting in a complete loss of cPLA_2α_ protein expression and a profound inability of whole blood, isolated platelets, peripheral blood monocytes, or blood outgrowth endothelial cells to release eicosanoids and reduced levels of urinary markers of prostacyclin and thromboxane.^34^ The clinical, genetic, and phenotypic details of the patient are published elsewhere.^32^, ^33^, ^34^ The patient had a lifetime history of gastrointestinal disease with a diagnosis of cryptogenic multifocal ulcerous stenosing enteritis and was found to carry homozygous 4 bp deletion (g.155574_77delGTAA) in the *PLA2G4A* gene resulting in a frameshift of 10 amino acids before a premature stop codon (p.V707fsX10) and the loss of 43 amino acids (residues 707–749) at the C terminus of group IV A cPLA_2_α.^32^ Renal function declined because of tubulointerstitial nephritis (identified as xanthogranulomatous pyelonephritis on renal biopsy), leading to end-stage renal failure requiring dialysis.

Blood was collected into heparinized tubes by venipuncture, and plasma was separated by centrifugation from 9 healthy volunteers and the patient bearing the homozygous mutation in the *PLA2G4A* gene (8 samples pretransplant and 5 samples posttransplant). The pretransplant samples were taken from between 27 months, 24 days, and 1 month, and 19 days before the transplant. After the kidney transplant had stabilized, blood samples were collected for analysis at 1 to 6 months posttransplant. Studies were conducted in accordance with the principles of the Declaration of Helsinki after local ethical approval (healthy volunteers: St Thomas’s Hospital Research Ethics Committee, reference 07/Q0702/24; individual lacking cPLA_2α_: South East NHS Research Ethics Committee).

### Mouse Samples

Male and female, 6 to 8-week-old mice lacking COX-2^17^^,^^31^ or PGIS^22^ were used and compared to age-matched, sex-matched, and strain-matched wildtype controls. All animal experiments were conducted in line with the Animals (Scientific Procedures) Act 1986 (2013 revision) and EU directive 2010/63/EU. Procedures were reviewed and approved by the Shantou University Institutional Animal Research and Use Committee, the Animal Welfare Committee of the State Agency Darmstadt (Germany) and/or the Imperial College London Ethical Review Panel (PP1576048). Mice were euthanized by carbon dioxide narcosis delivered by inhalation, blood collected from the inferior vena cava into heparin (10 U/ml final; Leo Laboratories, UK) and plasma separated by centrifugation.

### Organotypic Kidney Slices

Mice were euthanized as above, exsanguinated, the vasculature flushed with sterile phosphate buffered saline, and both right and left kidneys collected into sterile phosphate buffered saline. For slice preparation (within 6 hours of tissue collection), whole kidneys were immobilized in agarose (2%) and 150 μm slices cut in the sagittal plane using a Compresstome VF-300-0Z vibrating microtome (Precisionary Instruments, USA). Slices were inspected to check their integrity and composition and any adherent agarose carefully removed. COX-2 expression is enriched in the medulla region of the kidney, although COX-2 is also expressed within the cortex.^5^^,^^35^ To capture as closely as possible key cellular locations of renal COX-2, only slices containing both medulla and cortex regions were used (Supplementary Figure S1). Each slice was placed into individual wells of a 48-well plate with 200μl of Dulbecco’s Modified Eagle’s Medium (Sigma, UK) supplemented with nonessential amino acids (Gibco, UK), Pen-strip (Sigma, UK) and L-glutamine (Sigma, UK) and slices incubated at 37°C in an atmosphere of 5% carbon dioxide. After 1 hour equilibration period, the media were discarded and replaced, and slices incubated for a further 24 or 72 hours before collection of conditioned media for analysis. For each condition, duplicate kidney slices were studied, and measurements averaged. In these studies, individual (left and right) kidneys were considered as separate n values.

### Measurement of Analytes

ADMA, arginine, and citrulline were measured, within a panel of amines, in human and mouse plasma by ultra-high performance liquid-chromatography tandem mass spectrometry following derivatization with AccQTag as described previously.^36^ ADMA was below the limit of detection in 2 of 55 samples and inputted at the assay limit of quantification. ADMA was also measured in the same samples using enzyme-linked immunosorbent assay (ELISA). from DLD Diagnostika (Germany) according to manufacturer’s instructions. Mouse cystatin C was measured using a DuoSet ELISA from R&D Systems (Abingdon, UK) according to manufacturer’s instructions (1/2000 dilution). Human cystatin C was measured using a LEGENDplex bead capture immunoassay (Biolegend, UK) according to manufacturer’s instructions (1/50 dilution) with data acquired on a LSRFortessa II flow cytometer (BD Biosciences, UK).

### Statistics

Unless otherwise indicated data are presented as individual points relating to samples from separate animals or separate healthy donors or repeat collections, on separate days, from the patient carrying a mutation in the *PLA2G4A* gene. Analysis was performed using Prism V9 software (GraphPad Software, Boston, MA). Statistical tests are described in the figure legends and a *P*-value < 0.05 is considered statistically significant.

## Results

**Figure 1:**
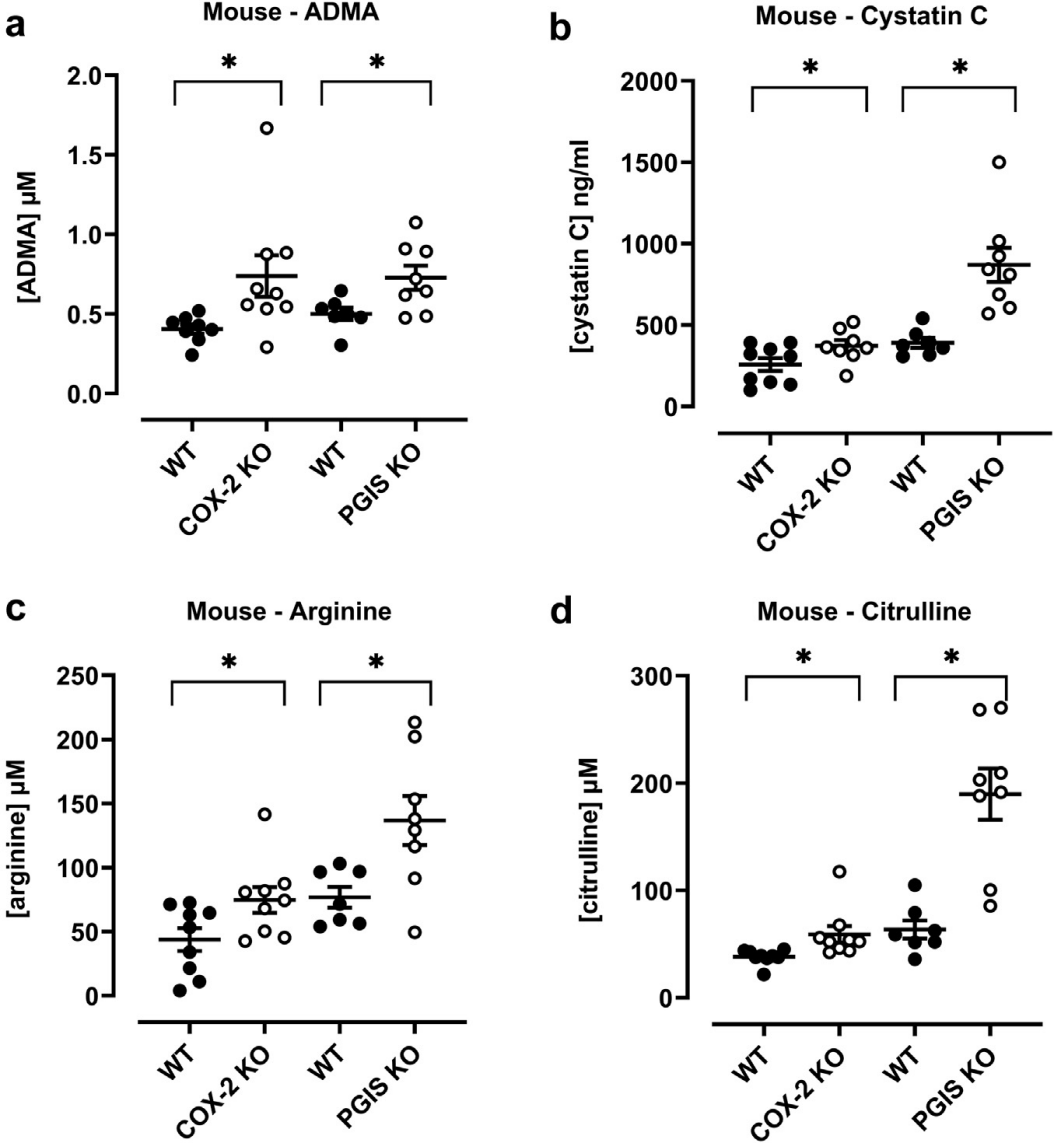
Plasma ADMA (a), cystatin C (b), arginine (c), and citrulline (d) in mice lacking COX-2/prostacyclin (PGIS) synthase. Data are mean ± SEM for *n* = 8/9 (COX-2 KO/WT); *n* = 7/8 (PGIS KO/WT) analyzed by unpaired t-tests. ∗*P* < 0.05. ADMA, asymmetric dimethylarginine.

In agreement with our previous work, germline, global loss of COX-2,^17^ or PGIS^22^ resulted in significant increases in plasma ADMA measured using ultra-high performance liquid-chromatography tandem mass spectrometry (Figure 1). Changes in ADMA levels were validated using ELISA for samples from COX-2 knockout mice (wildtype, 0.60 ± 0.101 μM: COX-2 knockout 0.88±0.09 μM) and PGIS knockout mice.^22^ Our group^17^ and others have shown that genetic deletion of COX-2^15^^,^^16^ or PGIS^37^ in mice results in compromised renal morphology and/or function, corroborating the critical role that COX-2 derived prostacyclin has in kidney homeostasis. Similarly, in the current study where renal function was assessed by plasma levels of cystatin C, we found compromise in genetically modified mice lacking either COX-2 or PGIS. Moreover, ADMA showed significant positive correlations with cystatin C in mouse model samples (*r* = 0.39, *P* = 0.03; Table 1).

**Table 1:** Pearson correlation parameters *r* (Pearson Correlation Coefficient) and *P*-values for analysis between cystatin C and amino acids (ADMA, Arginine and Citrulline)

Analyte	Mice		Human	
	*r*	*P*	*r*	*P*
ADMA	0.3941	0.0282	0.4996	0.0179
Arginine	0.5809	0.0005	−0.1745	0.4373
Citrulline	0.7363	<0.0001	0.9304	<0.0001

ADMA, asymmetric dimethylarginine.

**Figure 2:**
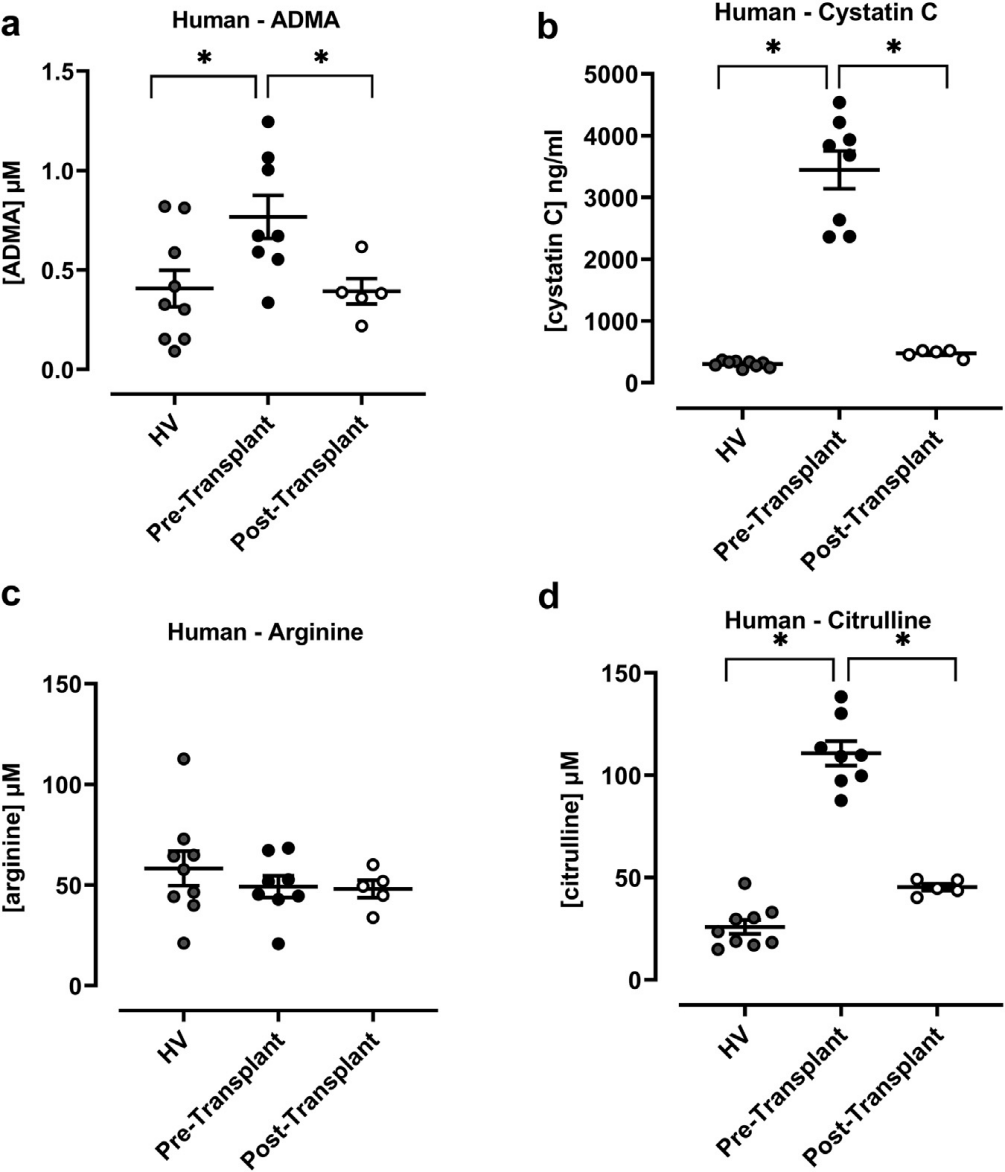
Plasma ADMA (a), cystatin C (b), arginine (c), and citrulline (d) in samples from human subjects with or without renal prostanoid synthetic capacity. Data from plasma samples of healthy volunteers (HV) or from a patient carrying a homozygous 4 bp deletion (g.155574_77delGTAA) in the *PLA2G4A* gene resulting in a complete loss of cPLA_2_α protein expression and profound reductions in the generation of eicosanoids before (Pretransplant) and after (Posttransplant) receiving a genetically normal kidney which restored renal prostanoid production.^34^ Data are mean ± SEM for *n* = 9 (HV); *n* = 8 (Pretransplant); *n* = 5 (Posttransplant) analyzed by 1-way ANOVA followed by Dunnett's multiple comparisons test. ∗*P* < 0.05. ADMA, asymmetric dimethylarginine.

All mice samples were analyzed together and derived from data in Figures 1 and 2.

Next, we measured ADMA and cystatin C in plasma samples from healthy volunteers and from a patient carrying a loss of function mutation in cPLA_2_ resulting in global loss of COX activity. Samples were measured before and after the patient received a genetically normal kidney. After the transplant, the patient retained an inability to produce prostanoids systemically but gained renal COX function.^34^ Further details describing the clinical characteristics of this patient^32^, ^33^, ^34^ and levels of prostanoids^34^ from the samples used in this study are described elsewhere. Similar to results in mice lacking COX-2 or PGIS, we found significant increases in plasma ADMA in samples from the patient pretransplant compared to levels in healthy volunteers. Renal function, assessed by cystatin C levels, was decreased in the patient pretransplant compared to healthy volunteers and restored after their kidney transplant (Figure 2). This agrees with the clinical scenario and our previous reported levels of plasma urea and creatinine in this individual.^34^ In line with this, we found that in samples from human subjects, levels of ADMA directly correlated with cystatin C (Table 1). Similar levels of ADMA were reported by Claes *et al.*^38^ who analyzed samples from incident renal transplant recipients at the time of transplant and at 3 and 12 months after transplant. In their study, ADMA declined from 0.63μM before transplant to 0.55 μM at 12 months posttransplant, although in their study levels did not normalize entirely (i.e. compared to control values).

**Figure 3:**
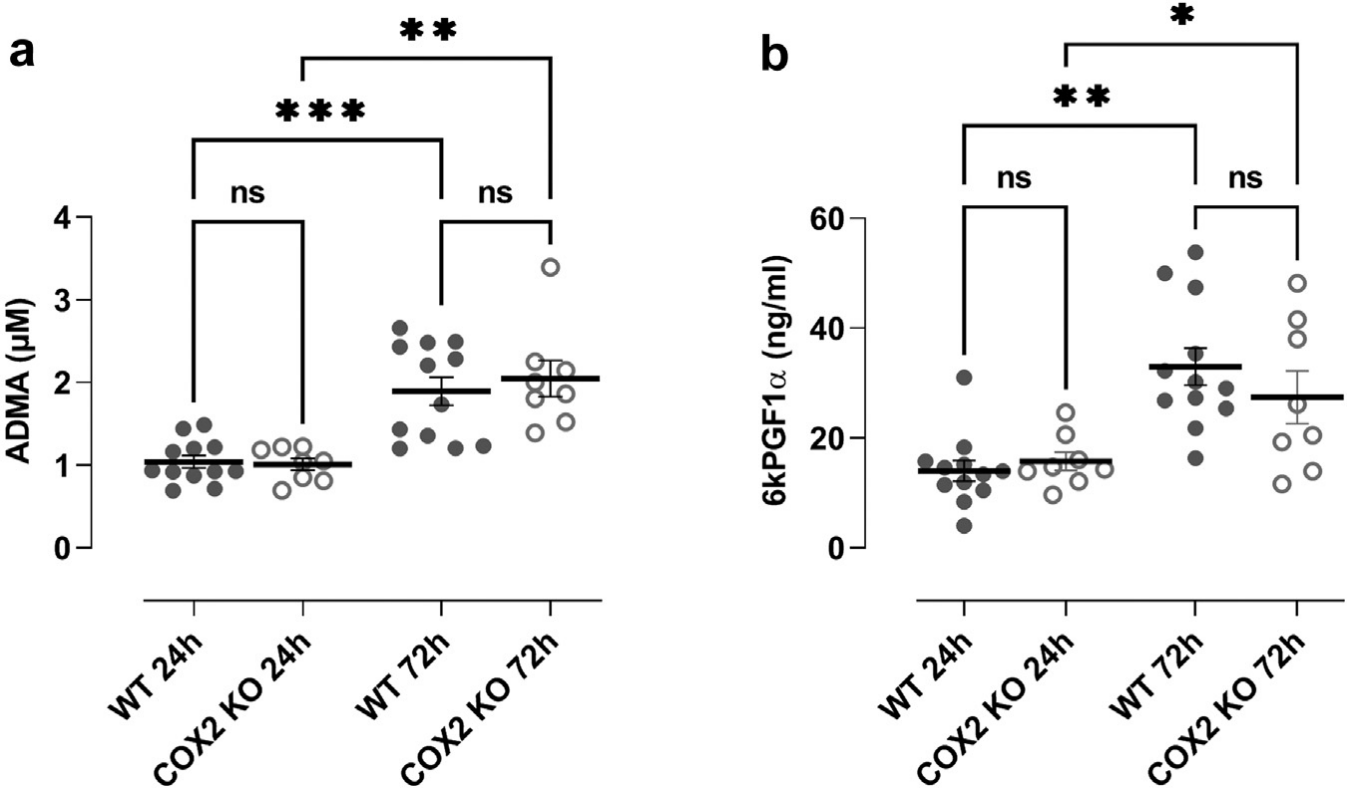
Release of ADMA (a) and prostacyclin (b) by organotypic kidney slices from wild type (WT) and COX-2 knockout (COX-2 KO) mice. The figure shows individual data points and mean ±SEM for *n* = 12/8 (COX-2 KO/WT) kidneys. Data were analyzed using 1-way ANOVA followed by Tukey's multiple comparisons test. ∗*P* < 0.05. ADMA, asymmetric dimethylarginine.

The modest magnitude of correlation between ADMA and renal function may be explained by the degree of complexity in the biological pathways involved in the synthesis, metabolism, and excretion of ADMA. ADMA is metabolized by intracellular DDAH and a component is excreted in urine. DDAH levels are reduced in germline COX-2 knockout mice^17^ but not in conditional knockout models,^30^ possibly because of renal dysfunction and oxidative stress, whereas in chronic kidney disease, DDAH levels are reduced because of loss of renal mass.^39^ To further understand metabolic contribution of COX-2 derived prostacyclin and methylarginines, we performed *ex vivo* studies using organotypic kidney slices incubated in culture for 24 to 72 hours. Conditioned media from kidney slices released ADMA and prostacyclin at 24 hours, which was increased at 72 hours (Figure 3). No difference was seen in levels of ADMA or prostacyclin released *in vitro* from kidney slices from COX-2 knockout mice compared to wildtype controls. On the face of it, these results suggest that increases in ADMA seen in plasma are primarily a result of renal clearance. However, it should be noted that although using intact kidney slices has the advantage of including gross renal tissue and therefore capture all cell types, it has the disadvantage of potentially missing events in specific regions and a loss of signaling compartmentalization. This limitation explains the lack of effect of COX-2 deletion on prostacyclin release. As detailed above, COX-2 in the kidney is critically important, but it is expressed in highly localized regions whereas COX-1 predominates throughout.^40^^,^^41^ It should also be noted that metabolic processes may be influenced as a direct result of tissue culture.

ADMA is a competitive inhibitor of eNOS. In addition to prostacyclin, endothelial derived nitric oxide protects the kidney as a well-established vasodilator.^42^ Through increased levels of inhibitory ADMA, it is therefore conceivable that loss of renal COX-2/prostacyclin drives renal dysfunction by a combination of reduced prostacyclin and reduced eNOS activity. As a competitive inhibitor, the potency of ADMA against eNOS is directly proportionate to the levels of the available substrate, arginine. Arginine is a semiessential amino acid and can be generated within endothelial cells^43^, ^44^, ^45^, ^46^, ^47^, ^48^ from citrulline via the urea cycle. In our study, plasma concentrations of arginine and citrulline were increased in both COX-2 and PGIS knockout mice (Figure 1), whereas citrulline (but not arginine) was increased in plasma samples from the patient before their kidney transplant (Figure 2). Nevertheless, it is not possible to conclude from measuring plasma levels alone, that either (i) increased ADMA is functionally important on the eNOS system in our study or that (ii) any effects of increased methylarginine on eNOS activity are mitigated by elevated substrate. This is because it is the concentration of free ADMA and other endogenous inhibitors versus the concentration of free arginine within cells that dictates eNOS activity. Levels of intracellular methylarginines and arginine are influenced not only by circulating levels of the amine but also by intracellular metabolism and uptake mechanisms.^49^

## Discussion

COX-2 protects the cardiorenal system and though the mechanisms remain to be fully established, restraining ADMA is a plausible contributory pathway. Here, we confirm previous observations using mouse samples and report novel findings about an individual lacking COX-2 activity secondary to a loss of function mutation in cPLA_2_, before and after receiving a donor kidney. Our findings corroborate the idea that COX-2 and prostacyclin are critical regulators of renal function and that elevations in ADMA are directly linked to renal impairment. These findings are in line with the recognized role that the kidney COX-2 plays in cardiorenal protection and highlights the potential importance of the kidney dysfunction in cardiovascular side effects of NSAIDs.

## Disclosure

JAM and TDW have received consultancy fees and acted as an expert witness. JAM is on the advisory board for Antibe and owns shares in Antibe Therapeutics Inc. The other authors declared no competing interests.

## Supplementary Material

Supplementary File (PDF)

**Figure S1.** Image showing typical kidney slice containing intact pelvis, medulla, and cortex regions.
