# Supplementary figures and images for: Renal Function Underpins the Cyclooxygenase-2: Asymmetric Dimethylarginine Axis in Mouse and Man
Source: Kidney Int Rep. 2023 Mar 23;8(6):1231–8. doi: 10.1016/j.ekir.2023.03.014 (PMC10239776; doi:10.1016/j.ekir.2023.03.014)

## Typical kidney slice

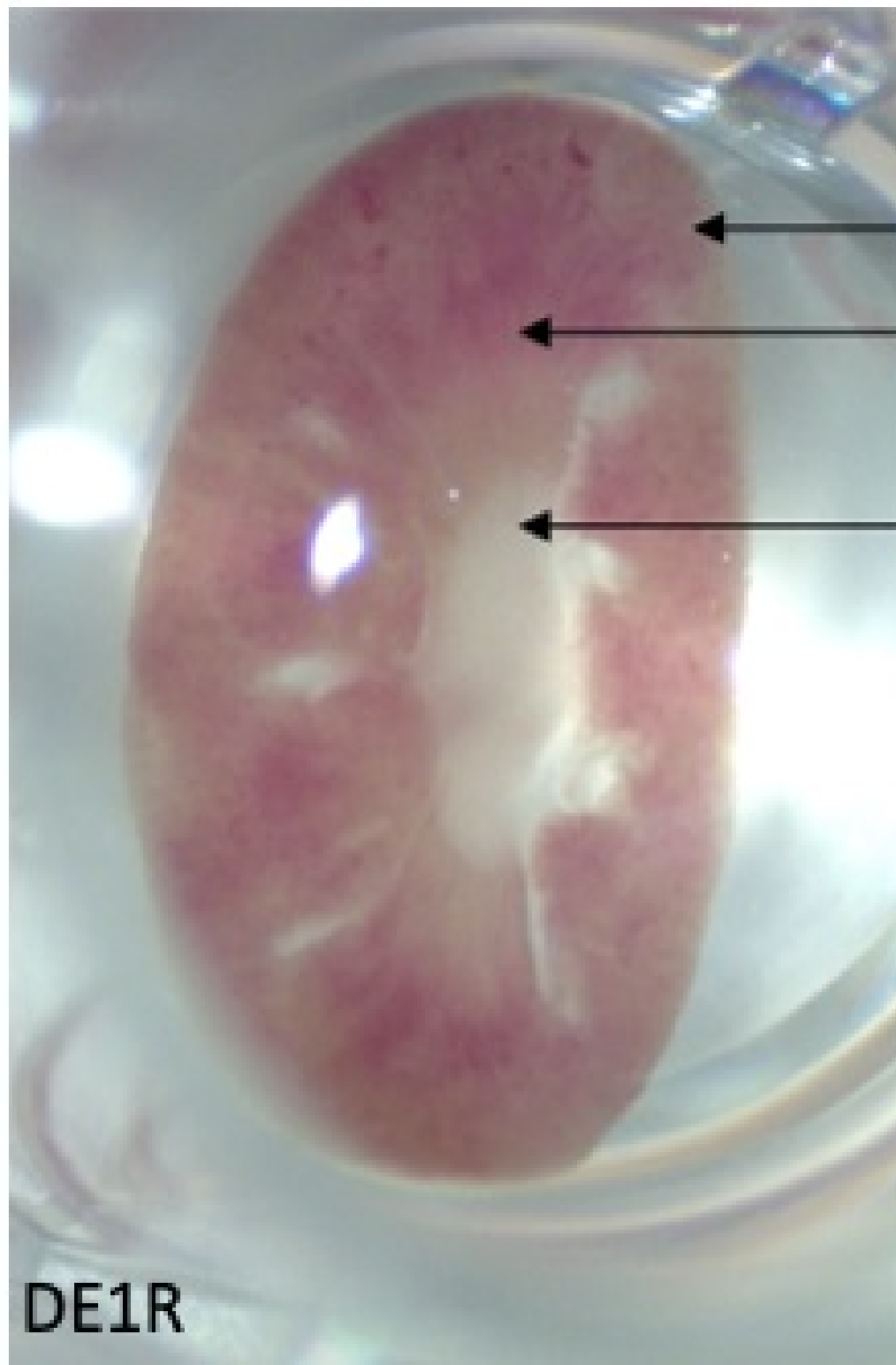

cortex  
medulla  
pelvis

DE1R

Supplement: Supplementary File (PDF) [file mmc1.pdf]
